# Liver kinase B1 expression is associated with improved prognosis and tumor immune microenvironment features in small cell lung cancer

**DOI:** 10.3389/fonc.2025.1552506

**Published:** 2025-04-04

**Authors:** Alessandro Dal Maso, Federica Ferrarini, Giovanni Esposito, Sonia Anna Minuzzo, Anna Maria Puggia, Federica Pezzuto, Elisabetta Zulato, Loc Carlo Bao, Mattia De Nuzzo, Alessandra Ferro, Stefano Frega, Giulia Pasello, Fiorella Calabrese, Matteo Fassan, Federico Rea, Valentina Guarneri, Stefano Indraccolo, Laura Bonanno

**Affiliations:** ^1^ Medical Oncology 2, Veneto Institute of Oncology IOV - IRCCS, Padova, Italy; ^2^ Basic and Translational Oncology, Veneto Institute of Oncology IOV - IRCCS, Padova, Italy; ^3^ Immunology and Molecular Oncology Diagnostics, Veneto Institute of Oncology IOV - IRCCS, Padova, Italy; ^4^ Department of Surgery, Oncology and Gastroenterology, University of Padova, Padova, Italy; ^5^ Anatomy and Pathological Histology, Veneto Institute of Oncology IOV - IRCCS, Castelfranco Veneto, Italy; ^6^ Department of Cardiac, Thoracic, Vascular Sciences and Public Health, University of Padova, Padova, Italy; ^7^ Department of Medicine, University of Padua, Padua, Italy; ^8^ Veneto Institute of Oncology IOV - IRCCS, Padua, Italy; ^9^ Thoracic Surgery Unit, Padova University Hospital, Padova, Italy

**Keywords:** small cell lung cancer, liver kinase B1, serine/threonine kinase 11, immunohistochemistry, overall survival, tumor immune microenvironment

## Abstract

**Background:**

Small cell lung cancer (SCLC) is characterized by early metastatic potential and poor prognosis. Liver kinase B1 (LKB1) is a tumor suppressor and a cell metabolism regulator. LKB1 downregulation has been associated with a cold tumor immune microenvironment (TIME). We aimed to analyze the role of LKB1 in SCLC in relation to its association with overall survival (OS) and TIME components.

**Methods:**

We retrospectively evaluated SCLC patients consecutively treated at our institution from 1996 to 2020 with available tissue. LKB1, PD-L1 on tumor cells and on tumor immune-infiltrating cells, CD8, and FOXP3 were evaluated by immunohistochemistry (IHC), categorized according to predefined cutoffs. The primary endpoint was the description of LKB1 expression, and the secondary endpoints were the association with prognosis and TIME features.

**Results:**

Tissue samples of 138 out of 481 SCLCs were adequate for molecular analyses. Eighty patients had limited stage (LS) at diagnosis and 58 had extended stage (ES). The median LKB1 IHC score was 4. Patients with IHC score >4 (*n* = 67) were classified as LKB1-positive. The probability of LKB1 positivity was higher in LS [odds ratio 2.78, 95% confidence interval (95% CI) 1.18–7.14]. At the data cutoff (2 January 2024), 123 patients died. The median OS (mOS) was 14.0 months (95% CI 11.5–19.4). mOS was significantly longer in patients with LKB1-positive expression [32.4 months (95% CI 13.6–62.4) vs. 11.2 months (95% CI 8.7–14.7); *p* < 0.001]. At multivariate analysis, positive LKB1 expression, LS, and no weight loss at diagnosis were confirmed as independent positive prognostic factors. TIME features were evaluated in 70 patients. Unexpectedly, LKB1-negative samples were more likely to show CD8^+^ tumor-infiltrating lymphocytes (TILs; *p* = 0.013). No association with PD-L1 expression nor the presence of FOXP3^+^ TILs was found.

**Conclusion:**

LKB1 expression is a potential positive prognostic marker in SCLC. In this series, LKB1 expression was negatively associated with the presence of CD8^+^ TILs.

## 1 Introduction

Lung cancer represents the leading cause of cancer-related deaths worldwide (1). Small cell lung cancer (SCLC) accounts for nearly 15% of lung cancer diagnoses and is a high-grade neuroendocrine cancer, associated with a dismal prognosis (2). Limited stage (LS) SCLC is defined as a tumor confined to one hemithorax and regional lymph nodes, eligible for curative intent treatment. Radical intent radiotherapy (RT) and concurrent chemotherapy (ChT) are the standard of care for LS, although, in selected cases, surgery followed by adjuvant platinum and etoposide (PE) is considered, in the absence of lymph node involvement at clinical staging (2, 3). The upfront treatment of extended stage (ES) SCLC has been unchanged for decades and consisted of PE ChT (2, 3). Recently, several randomized phase III trials demonstrated the superiority of first-line chemoimmunotherapy, showing a similar rate of overall survival (OS) improvement versus standard ChT alone (3–10). A comparable benefit was shown in real-world studies including patients with relevant comorbidities, older age, and high-symptom burden (11, 12). Despite a statistically significant difference, clinical outcome improvement is only modest and only approximately 15% are alive after 3 years of chemoimmunotherapy treatment (5, 7, 13).

The study of the molecular features of SCLC is one key element in order to finally improve clinical outcome (14). Recently, transcriptomic profiling has led to a new molecular classification of SCLC, based on the deregulation of key cellular pathways. No clear prognostic impact was found for the classification, whereas an inflamed subtype, characterized by overexpression of human leukocyte antigens, immune checkpoints, and cytokines, was suggested as a potential positive predictive factor for chemoimmunotherapy (15). Conversely, the SCLC-N subtype was characterized by overexpression of the *NEUROD1* transcription factor, and SCLC-N cell lines were highly sensitive to multiple aurora kinase (AURK) inhibitors (15). AURK is a serine/threonine kinase localized to the centrosome, which is crucial during the cell cycle to guarantee the right chromosome segregation, but has also tumor-promoting roles unrelated to mitosis (16–19). AURK has been involved in phosphorylating liver kinase B1 (LKB1, also known as serine/threonine kinase 11, STK11) resulting in the suppression of the LKB1/adenosine monophosphate-activated protein kinase (AMPK) signaling pathway and the proliferation, invasion, and migration of non-small cell lung cancer (NSCLC) cells (20).

LKB1 is a tumor suppressor and cell metabolism regulator and has been shown to modulate tumor immune microenvironment (TIME) features in NSCLC (21–28). *LKB1* alterations have been described in approximately 30% of NSCLCs, including nonsense mutations, frameshift mutations, large exonic deletions, and intronic mutations in conserved splice sites (29, 30). These mutations translate into either a truncated, inactive protein or the absence of the protein. Epigenetic inactivation has also been demonstrated, with promoter hypermethylation detected in up to 13% of the specimens (31, 32).

Few data are available for *LKB1* alterations in SCLC and mostly concerning *LKB1* mutations (33–36). Based on these premises, we aimed to describe LKB1 expression in SCLC and investigate its potential prognostic role and association with TIME features.

## 2 Materials and methods

### 2.1 Study patients and endpoints

We retrospectively evaluated all SCLC cases consecutively treated at Veneto Institute of Oncology IOV - IRCCS with available tissue, from 1996 to 2020. The inclusion criteria were pathological diagnosis of SCLC, availability of clinical data and adequate follow-up, and availability of pathological samples.

Histological diagnosis was obtained by formalin-fixed paraffin-embedded (FFPE) samples that were collected before starting the primary treatment. Staging included brain, neck, chest, and abdomen computed tomography (CT) scan with iodine contrast, bone scan and, in selected cases, brain magnetic resonance imaging and/or total body ^18^F-fluorodeoxyglucose positron emission tomography/CT scans.

A multidisciplinary team, including dedicated medical oncologists, radiation oncologists, thoracic surgeons, pneumologists, a radiologist, and a nuclear medicine physician evaluated all the patients and decided on the treatment plan, according to guidelines. Patients with limited stage disease received radical chemoradiotherapy and, in selected cases, surgery followed by adjuvant ChT. ChT consisted of four to six PE cycles, while 3D conformal RT was started as early as possible. After upfront surgery, four cycles of adjuvant PE were administered, when clinically feasible. If the patient responded to radical treatment, prophylactic cranial irradiation was discussed with the patient. Extended SCLC cases received upfront ChT with PE (up to six cycles) and palliative RT was administered when indicated. ChT and RT were administered at Veneto Institute of Oncology IOV - IRCCS. Surgery was carried out at the Thoracic Surgery Unit of Padova University Hospital and histological diagnosis at the Pathology Unit of Padova University Hospital.

Clinical variables collected from electronic medical records were sex, age at diagnosis, smoking status, Eastern Cooperative Oncology Group (ECOG) performance status (PS) at diagnosis, presence of symptoms at diagnosis, presence of weight loss at diagnosis, stage at diagnosis, central nervous system (CNS) metastases at diagnosis, and primary treatment.

The Ethics Committee of Veneto Institute of Oncology IOV - IRCCS in Padova evaluated and approved the study and informed consent that was required, whenever feasible, for the collection, analysis, and publication of data, according to the Helsinki Declaration and Italian Data Protection Authority dispositions (approval number: IOV-MICRO-2017).

The primary endpoint of the study was the description of LKB1 expression by immunohistochemistry (IHC), and the secondary endpoints were the association with prognosis and TIME features.

### 2.2 Immunohistochemistry

Four-micrometer-thick FFPE tissue slices were used for IHC testing. IHC staining was performed automatically using the BOND RX system (Leica Biosystems, Nussloch, Germany).

The primary antibodies were as follows: Ley 37D/G6 (Santa Cruz Biotechnology, Dallas, TX, USA) for LKB1, C8/144B clone (DAKO, Carpinteria, CA, USA) for cluster of differentiation 8 (CD8), 236A/E7 clone (Abcam, Cambridge, UK) for forkhead box P3 (FOXP3), and 22C3 clone (DAKO, Carpinteria, CA, USA) for programmed death-ligand 1 (PD-L1). The semiquantitative score for LKB1 expression was calculated: the proportion of cell staining was scored 1 to 6 (0%–4%: 1, 5%–20%: 2, 21%–40%: 3, 41%–60%: 4, 61%–80%: 5, 81%–100%: 6), whereas intensity was scored 0 to 3+. The two scores were multiplied to obtain the final score (ranging from 0 to 18). The median LKB1 score was calculated and chosen as the cutoff for LKB1 expression positivity (37, 38).

TILs were described using semiquantitative criteria: absent or sporadic (0), moderate (1+), abundant (2+), and highly abundant (3+). A specimen was considered negative for CD8^+^ TILs presence when scoring 0–2+ and positive when scoring 3+, whereas a case was considered negative for FOXP3^+^ TILs when IHC scored 0 and positive when IHC scored 1–3+ (39). PD-L1 expression in TC and TIIC was considered negative when 0% and positive when ≥1% (39).

### 2.3 Statistical analysis

Qualitative clinical and biological variables were described with counts and proportions, whereas quantitative variables were described with median and range. Associations between qualitative variables were evaluated using chi-squared or Fisher exact test, as appropriate; odds ratios (ORs) were calculated along with the 95% confidence interval (95% CI), and multivariate analysis was performed with logistic regression. Kaplan–Meier survival curves were built to estimate median OS with 95% CI, and the log-rank test was used to compare survival curves. Cox regression analysis was carried out to estimate univariate and multivariate hazard ratios (HRs) for death, along with 95% CI. The significance level was set at *p <*0.05. Statistical analysis was performed with the R software, version 4.3.0 (The R Foundation, Vienna, Austria).

## 3 Results

### 3.1 Study population

All pathological samples were revised by an experienced pathologist, and 138 out of 481 cases were judged adequate for molecular testing and included in the analysis. The clinical features of the study population considered for analysis are summarized in **Table 1**. Eighty-seven of 138 patients (63.0%) were men and 51 (37.0%) were women, and the median age at diagnosis was 68 years (range: 47–82). One hundred and thirty-four patients (97.1%) were smokers, 125 (90.6%) had ECOG performance status (PS) ≤1, 99 (71.7%) had any symptom at diagnosis, and 24 (17.4%) had weight loss at diagnosis.

**Table 1:** Study population.

Variable	*n* = 138
Sex	
Male	87 (63.0%)
Female	51 (37.0%)
Age	
Median (range)	68 (47–82)
<70	81 (58.7%)
≥70	57 (41.3%)
Smoker	
Yes (current/former)	134 (97.1%)
No	4 (2.9%)
ECOG PS	
≤1	125 (90.6%)
>1	13 (9.4%)
Symptoms at diagnosis	
Yes	99 (71.7%)
No	39 (28.3%)
Weight loss at diagnosis	
Yes	24 (17.4%)
No	114 (82.6%)
Stage at diagnosis	
Limited	80 (58.0%)
Extended	58 (42.0%)
CNS metastases at diagnosis	13 (22.4%)
Treatment	
Surgery followed by adjuvant ChT	49 (35.5%)
Concurrent ChT-RT	31 (22.5%)
Palliative ChT	58 (42.0%)

ECOG PS, Eastern Cooperative Oncology Group performance status; CNS, central nervous system; ChT, chemotherapy; RT, radiotherapy.

Eighty (58.0%) patients had LS disease at diagnosis: 31 (22.5%) were treated with radical RT concomitantly with ChT and 49 (35.5%) received surgery followed by adjuvant ChT. Fifty-eight (42.0%) patients had ES disease and received chemotherapy. Thirteen of 58 (22.4%) patients with ES had CNS metastases at diagnosis.

### 3.2 LKB1 expression and association with clinical features

**Figure 1:**
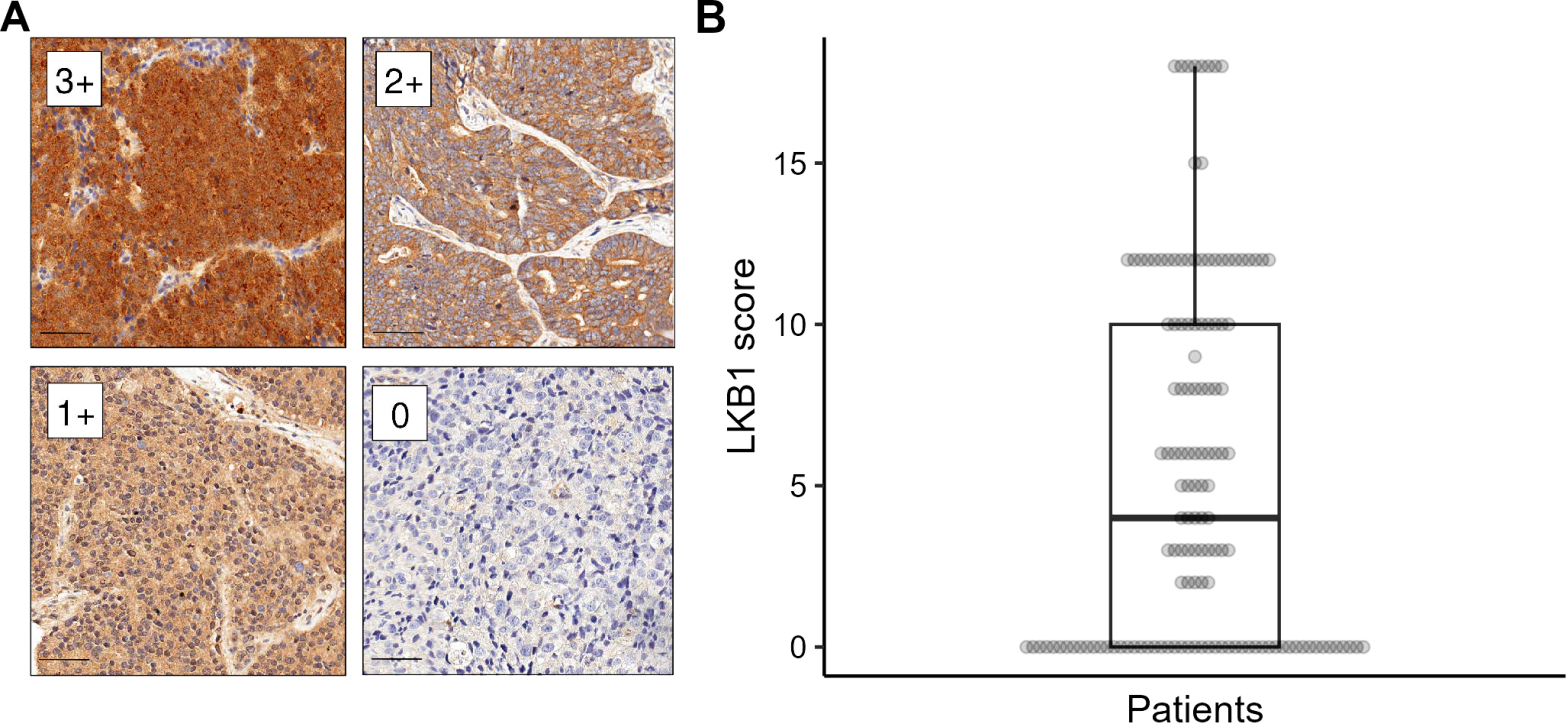
LKB1 immunohistochemistry (IHC). **(A)** Different levels of LKB1 IHC staining intensity (labeled as 0–3+) magnified at ×200, scale bar 50 µm. **(B)** Distribution of LKB1 IHC expression score in the study population.

LKB1 IHC was performed in all cases considered adequate for molecular analyses. In our series, the staining for LKB1 was localized exclusively in the cytoplasm of tumor cells, with varying intensities observed (**Figure 1**). The median LKB1 IHC score was 4 (range: 0–18; first quartile: 0, third quartile: 10). The median score was chosen as a positivity cutoff for LKB1 expression: 67 (48.5%) cases scored >4 and were considered positive, whereas 71 (51.5%) cases scored ≤4 and were considered negative.

We evaluated the association between LKB1 expression and clinical features, i.e., sex, age at diagnosis, smoking status, PS at diagnosis, symptoms at diagnosis, weight loss at diagnosis, and stage at diagnosis (**Table 2**). At univariate analysis, LKB1 expression was significantly correlated with PS ≤1 and LS disease. LKB1 expression was positive in 47 out of 80 (58.8%) patients with limited SCLC and in 20 out of 58 (34.5%) patients with extended SCLC. Multivariate analysis confirmed a significant positive association of LKB1 expression only with LS disease (OR 2.78, 95% CI 1.18–7.14, *p* = 0.023) (**Table 2**).

**Table 2:** Association between LKB1 expression and clinical features.

Variable		LKB1 > 4	LKB1 ≤ 4	Univariate OR (95% CI)	Multivariate OR (95% CI)
Sex	Male	45 (51.7)	42 (48.3)	1.41 (0.71–2.85, *p* = 0.331)	–
	Female	22 (43.1)	29 (56.9)	–	–
Age	<70	37 (45.7)	44 (54.3)	0.76 (0.38–1.49, *p* = 0.421)	–
	≥70	30 (52.6)	27 (47.4)	–	–
Smoker	Yes	64 (47.8)	70 (52.2)	0.30 (0.01–2.45, *p* = 0.309)	–
	No	3 (75.0)	1 (25.0)	–	–
ECOG PS	≤1	65 (52.0)	60 (48.0)	5.96 (1.52–39.53, ***p* = 0.024**)	4.57 (0.98–34.14, *p* = 0.080)
	>1	2 (15.4)	11 (84.6)	–	–
Symptoms at diagnosis	Yes	46 (46.5)	53 (53.5)	0.74 (0.35–1.56, *p* = 0.435)	–
	No	21 (53.8)	18 (46.2)	–	–
Weight loss at diagnosis	Yes	12 (50.0)	12 (50.0)	1.07 (0.44–2.61, *p* = 0.876)	–
	No	55 (48.2)	59 (51.8)	–	–
Stage at diagnosis	Limited	47 (58.8)	33 (41.2)	2.70 (1.37–5.55, ***p* = 0.005**)	2.78 (1.18–7.14, ***p* = 0.023**)
	Extended	20 (34.5)	38 (65.5)	–	–

OR, odds ratio; ECOG PS, Eastern Cooperative Oncology Group performance status. Bold text represents statistically significant differences.

### 3.3 LKB1 expression and association with prognosis

**Figure 2:**
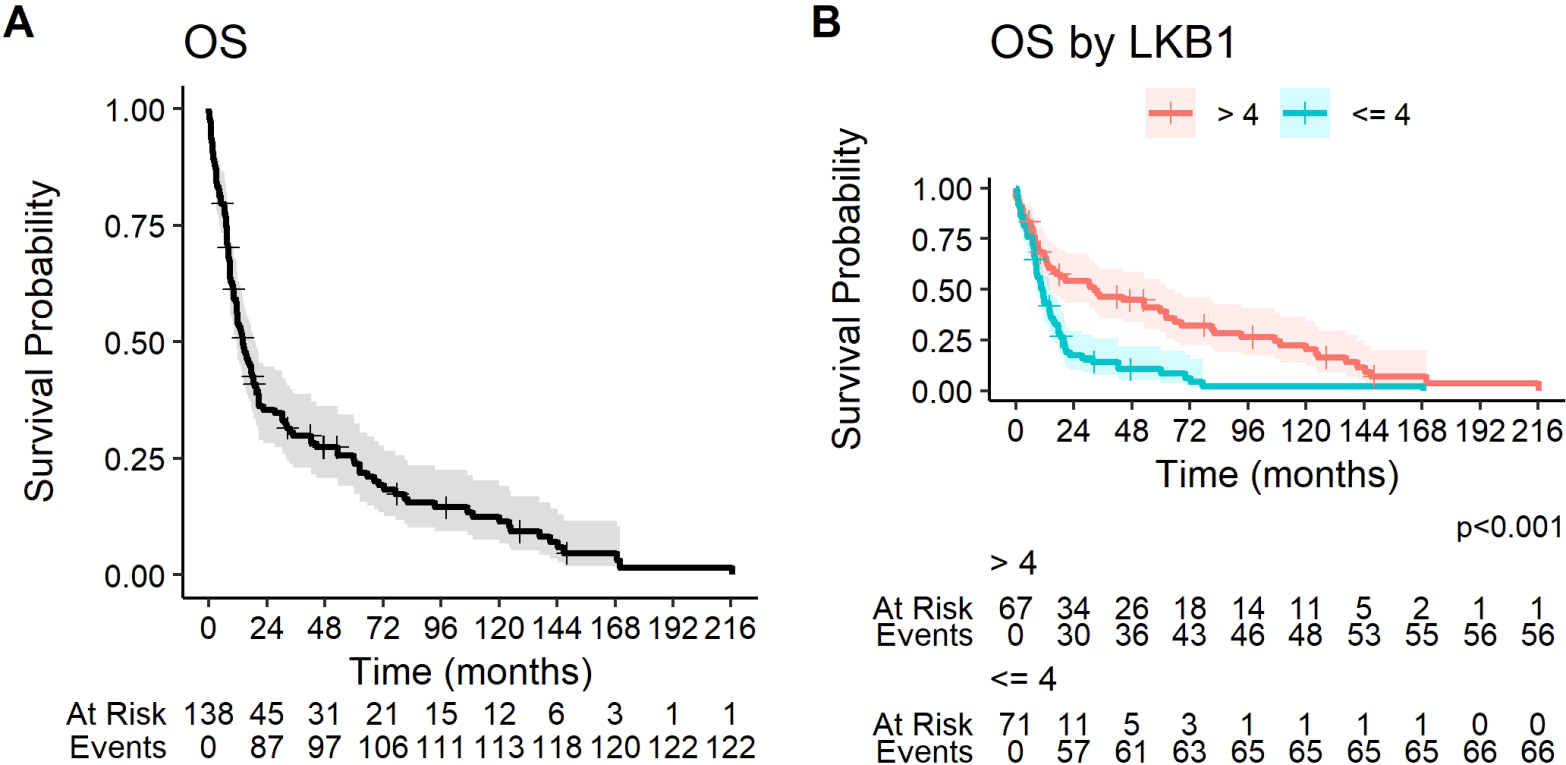
Kaplan–Meier curves for overall survival **(A)** in the overall study population and **(B)** according to LKB1 IHC expression.

At the data cutoff (2 January 2024), 123 patients (89.1%) had a death event. The median OS of the study population was 14.0 months (95% CI 11.5–19.4, **Figure 2**).

At univariate analysis, PS >1, weight loss, symptoms at diagnosis, female sex, and ES disease were significantly correlated to a worse prognosis (**Table 3**). Positive LKB1 expression was significantly associated with better prognosis: median OS was significantly longer in patients with LKB1 expression >4 (32.4 months, 95% CI 13.6–62.4 vs. 11.2 months, 95% CI 8.7–14.7, *p* < 0.001) (**Figure 2**).

**Table 3:** Survival analysis in the study population.

Variable		mOS, months (95% CI)	*p* (log rank)	Univariate HR (95% CI)	*p*	Multivariate HR (95% CI)	*p*
PS	>1	7.9 (3.1–NC)	**0.007**	2.28 (1.24–2.28)	**0.007**	1.83 (0.90–3.75)	0.096
	≤1	14.7 (11.9–20.6)					
Weight loss at diagnosis	No	16.3 (12.7–27.4)	**0.001**	0.48 (0.30–0.75)	**0.001**	0.57 (0.33–0.97)	**0.038**
	Yes	8.2 (4.9–13.6)					
Symptoms at diagnosis	No	60.2 (30.5–77.2)	**<0.001**	0.45 (0.30–0.69)	**<0.001**	0.72 (0.45–1.13)	0.161
	Yes	10.1 (8.1–13.0)					
Sex	Female	11.9 (8.4–17.6)	**0.032**	1.50 (1.03–2.18)	**0.033**	1.30 (0.87–1.95)	0.198
	Male	16.9 (11.7–31.7)					
Age	≥70	12.7 (8.2–20.8)	0.700	1.07 (0.74–1.55)	0.705	–	
	<70	14.7 (11.5–22.4)					
LKB1	≤4	11.2 (8.7–14.7)	**<0.001**	2.22 (1.52–3.26)	**<0.001**	1.73 (1.15–2.61)	**0.008**
	>4	32.4 (13.6–62.4)					
Stage at diagnosis	Extended	7.8 (6.7–10.1)	**<0.001**	4.82 (3.15–7.37)	**<0.001**	3.65 (2.24–5.94)	**<0.001**
	Limited	32.4 (20.3–65.7)					

HR, hazard ratio; 95% CI, 95% confidence interval; mOS, median overall survival. Bold text represents statistically significant differences.

At multivariate analysis, positive LKB1 expression, LS at diagnosis, and the absence of weight loss at diagnosis were confirmed as independent positive prognostic factors (**Table 3**).

### 3.4 TIME subset: clinical features and molecular results

We evaluated TIME features in 20 of 138 cases (TIME subset), due to the limited availability of the specimens. In this subset, 48 (68.6%) patients were men and 22 (31.4%) were women, and the median age at diagnosis was 68 years (range: 47–81). Sixty-seven (95.7%) patients were smokers, all had PS ≤1, 47 (67.1%) had any symptom at diagnosis, and 10 (14.3%) had weight loss at diagnosis. Fifty-four (77.1%) patients were diagnosed with LS disease, of which 45 (64.3%) were treated with surgery and 9 (12.9%) with chemoradiotherapy; 16 patients (22.9%) had ES at diagnosis and were administered ChT (**Supplementary Table S1**).

TIME features were investigated by IHC. PD-L1 expression on TCs was positive in 23 out of 70 cases (32.9%), whereas PD-L1 expression on TIIC was positive in 35 cases (50.0%). Nine (12.9%) cases were deemed positive (3+) for CD8^+^ TIL presence, and 54 cases (77.1%) were positive (1–3+) for FOXP3^+^ TILs (**Supplementary Table S2**).

We investigated the association between TIME features and clinical features. No TIME feature significantly correlated with sex, age, smoking status, PS, symptoms at diagnosis, weight loss, or stage at diagnosis (**Supplementary Table S3**).

We further explored the association between LKB1 expression and TIME features. A significant negative association was found between LKB1 expression and the presence of CD8^+^ TILs (OR 0.14, 95% CI 0.03–0.63, *p* = 0.013). No association was found between LKB1 expression and PD-L1 expression or the presence of FOXP3^+^ TILs (**Table 4**).

**Table 4:** Tumor immune microenvironment features and association with LKB1 expression.

Variable	LKB1 > 4 *n* = 50	LKB1 ≤ 4 *n* = 20	*p*
CD8^+^ TILs			**0.013**
0–2+	47 (94.0%)	14 (70.0%)	
3+	3 (6.0%)	6 (30.0%)	
FOXP3^+^ TILs			0.8
1–3+	39 (78.0%)	15 (75.0%)	
0	11 (22.0%)	5 (25.0%)	
PD-L1 TIIC			0.4
0%	35 (70.0%)	12 (60.0%)	
≥1%	15 (30.0%)	8 (40.0%)	
PD-L1 TC			>0.9
0%	25 (50.0%)	10 (50.0%)	
≥1%	25 (50.0%)	10 (50.0%)	

TILs, tumor-infiltrating lymphocytes; TIIC, tumor immune-infiltrating cells; TC, tumor cells. Bold text represents statistically significant differences.

At the data cutoff, 64 (91.4%) of 70 patients in the TIME subset had a death event. In the TIME subset, the median OS was 32.4 months (95% CI 16.3–62.4) (**Supplementary Figure S1**). At univariate analysis, the presence of weight loss, symptoms at diagnosis, and extended stage were significantly associated with a worse OS, while the presence of FOXP3^+^ TILs in tumor specimens associated with a significantly better OS. LKB1-positive expression showed a trend toward a better prognosis. Multivariate analysis confirmed FOXP3^+^ TILs and LS as independent positive prognostic factors (**Supplementary Figure S1**, **Supplementary Table S4**).

## 4 Discussion

SCLC is a recalcitrant cancer characterized by a dismal prognosis. The study of biomarkers in SCLC is highly challenging due to limited tissue availability, and currently, no predictive biomarker is available in clinical practice to tailor treatments.

In the depicted project, we explored the expression of LKB1 in SCLC and evaluated its prognostic role and relationship with TIME features. We chose to evaluate the potential biomarkers by using IHC, because the testing is relatively feasible, requires a relatively low amount of tissue, and accounts for the presence of *LKB1* epigenetic alterations (37, 40). In our cohort, LKB1 expression was deemed positive in 48.5% of cases. Limited data are available in the literature about LKB1 in SCLC. While no LKB1 mutations were reported in 11 SCLC cell lines (34, 35), a previous paper by Amin and colleagues reported a lower (13.3%) LKB1 IHC expression in 30 SCLC patients, of which 27 received surgery, but LKB1 expression was studied with a different antibody (D-19) and different scoring system (36). On the other hand, a recent retrospective work by Sivakumar and colleagues aimed at genotyping 3,600 SCLCs with FoundationOne CDx next-generation sequencing (NGS) panel, identified 62 *LKB1* mutant tumors, representing 1.7% of the entire cohort, which is a lower prevalence of LKB1 loss compared to our study. However, the prevalence of LKB1 loss may be underestimated because of the composition of the NGS panel and because the FoundationOne CDx panel does not account for epigenetic causes of LKB1 loss (33).

Our study identifies a strong association between LKB1 positivity and LS-SCLC. Given that LKB1 status was assessed prior to treatment, this association likely reflects inherent biological differences between LS and ES disease, which warrants further investigation.

In our study, we described significantly longer OS for patients expressing LKB1. This is consistent with the well-recognized LKB1 tumor suppressor functions and previous observations in NSCLC and other cancers (21, 27, 29, 30, 37). Importantly, in a previous broad molecular evaluation, Sivakumar and colleagues found only 7 *LKB1*-mutant patients out of 678, and a statistically significant worse overall survival for patients with *LKB1* mutations was reported, consistent with our results (33). Our data also confirm the prognostic significance of LKB1 impairment, demonstrating that its loss of expression is relatively frequent and can be detected in clinical practice using IHC, a simple and cost-effective method. Importantly, while our findings suggest LKB1 as a prognostic marker, we did not provide mechanistic validation through functional assays with SCLC cells, such as gene knockdown models, cytokine profiling, or immune response assays, as these were beyond the scope of our study. Nevertheless, the prognostic value of LKB1 in SCLC aligns with the well-established tumor suppressor functions of this gene, which encodes a master kinase that regulates cell migration, polarity, proliferation, and metabolism, primarily through downstream AMPK and AMPK-related kinase signaling, as extensively documented in prior studies (21, 41).

*LKB1* mutant/*KRAS* mutant NSCLCs showed significantly decreased tumor-associated macrophages, tumor-infiltrating lymphocytes, and PD-L1 expression and increased tumor-associated neutrophils compared to *KRAS* mutant only tumors (26). In order to understand the potential role in immunotherapy response, we focused on the expression of PD-L1, CD8^+^, and FOXP3^+^ TILs to study TIME. In a previous study by our team, consisting of 66 LS and 38 ES SCLCs, PD-L1 was expressed on TCs and TIICs in 25% and 40% of the cases, respectively. The proportion of PD-L1-positive cases was significantly higher in LS versus ES patients. CD8^+^ and FOXP3^+^ TILs were present in 59% and 72% of the samples, respectively. The presence of FOXP3^+^ TILs was associated with improved prognosis among LS patients, in univariate and multivariate analyses (39). In the present study, we were able to evaluate TIME features only in 50.7% of cases, due to limited tissue availability, but we confirmed the prognostic role of the FOXP3^+^ infiltrate. Positive cases of PD-L1 on tumor cells were 32.9%. The prevalence was similar to other larger studies with extended SCLCs (42, 43).

Moreover, we explored the association between LKB1 and TIME features. Previous studies described LKB1 as a promoter of a hot TIME in mouse models of NSCLC and other tumor types as well as clinical samples, with *LKB1* mutant tumors being characterized by decreased CD8^+^ TILs (26, 44, 45). However, in our cohort, LKB1 IHC positivity was negatively associated with the presence of CD8^+^ TILs. This aspect, partially contrasting the idea of LKB1 loss association with a cooler immune microenvironment, might be explained by a more complex role in modulating TIME and the involvement of TIME components. Interestingly, Best and colleagues showed that murine models of lung adenocarcinoma harboring *KRAS* and *LKB1* mutations exhibit increased glutaminase expression by tumor cells and increased glutamate in the tumor microenvironment, compared to *KRAS* mutant*/KEAP1* mutant lung adenocarcinomas. Glutamate abundance was associated with increased granzyme and interferon genes in *KRAS* mutant*/LKB1* mutant tumors, suggesting an increased activation of CD8^+^ cells (46). Whether this pathway could explain the negative association between LKB1 expression and CD8^+^ TILs in SCLC has to be elucidated. At the same time, a recent work by Qian and colleagues highlighted the role of lactate as the link between LKB1 metabolic roles and TIME modulation: in murine lung adenocarcinoma models, *LKB1* loss resulted in increased glycolysis and enhanced lactate production and export via the monocarboxylate transporter 4; this caused increased M2 macrophage polarization, which in turn resulted in hypofunctional T cells (47). Consistently, *LKB1* mutant lung adenocarcinomas from patients demonstrated a similar phenotype of enhanced M2 macrophage polarization and hypofunctional T cells (47). Further investigation of this pathway and M2 macrophage polarization in SCLC could explain our findings and should be warranted, especially considering that consolidation with immune checkpoint inhibitors would likely be the new standard of care in LS-SCLC, and in this context, it seems to have a higher impact on the outcome (48).

To the best of our knowledge, this is the first large series of SCLCs studied for the expression of LKB1. We were able to demonstrate that LKB1 impairment is likely to be associated with ES and worse prognosis, and its role in TIME modulation warrants further investigation.

The strengths of the study are the number of cases, particularly the high number of limited SCLC cases. This point is rather difficult to reach due to the clinical presentation of SCLC, and this allowed us to enhance the differential distribution of LKB1 expression according to stage. Finally, we were able to study LKB1 expression with IHC, which is a simple and inexpensive method, able to detect the consequences of mutational and also epigenetic *LKB1* alterations.

The weak points of the study are its retrospective nature, the lack of validation in a parallel cohort, and the lack of ES patients treated with chemoimmunotherapy combinations. Moreover, due to the limited availability of FFPE archival specimens, it was not possible to proceed with *LKB1* mutational analysis, metabolic profile analysis, and evaluation of their association with IHC status. Anyway, future analyses in patients treated with chemoimmunotherapy are ongoing, and parallel prospective tissue and liquid biopsy collection might be useful, in order to increase our knowledge about the role and pathway of LKB1 in SCLC.

## Data Availability

The raw data supporting the conclusions of this article will be made available by the authors, without undue reservation.
