# Supplementary material for: Liver kinase B1 expression is associated with improved prognosis and tumor immune microenvironment features in small cell lung cancer
Source: Front Oncol. 2025 Apr 4;15:1552506. doi: 10.3389/fonc.2025.1552506 (PMC12006004; doi:10.3389/fonc.2025.1552506)
Supplement: Supplementary file 1 [file DataSheet1.docx]

Supplementary Material

# Supplementary Figures


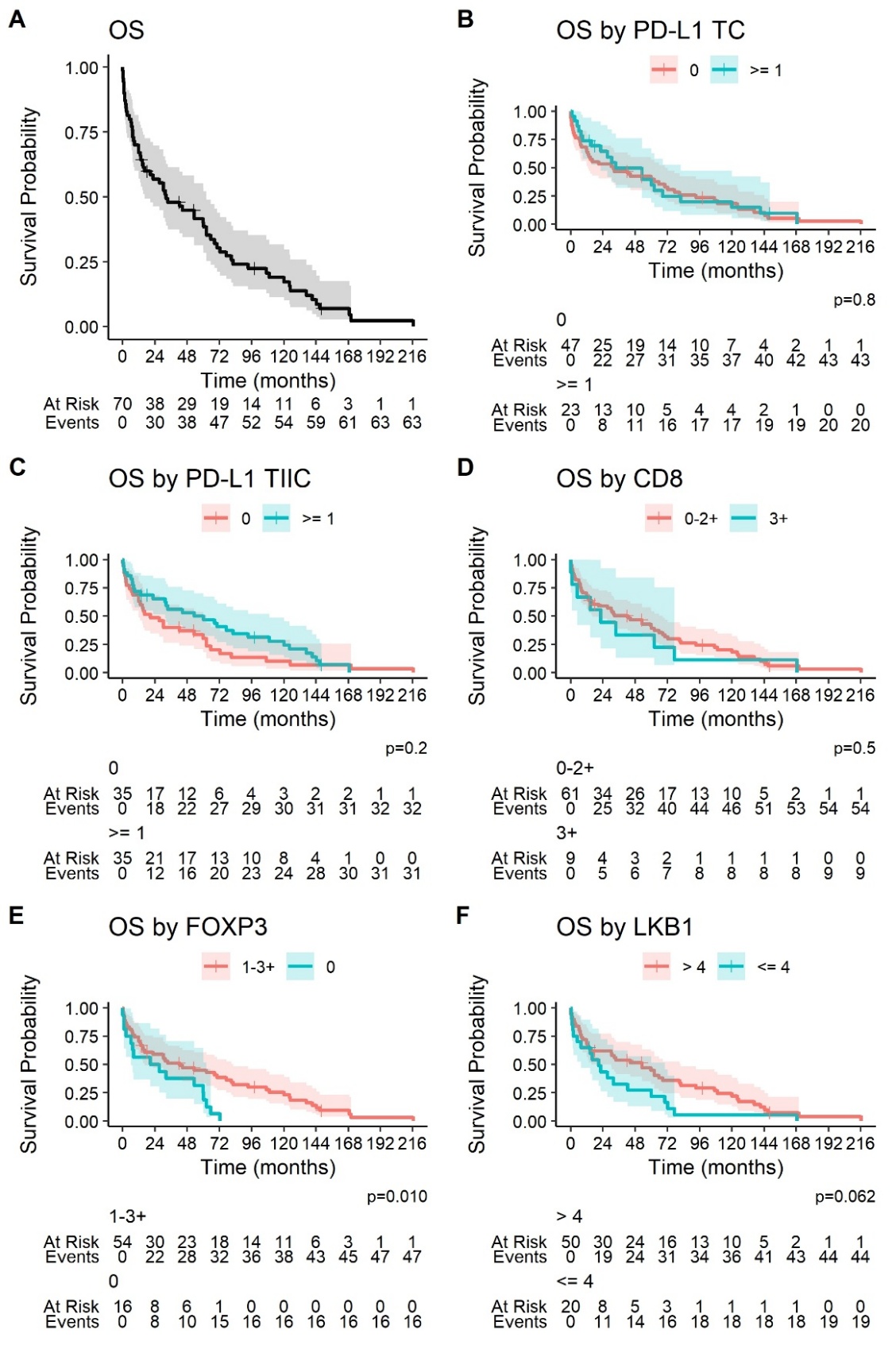


**Supplementary figure 1.** Kaplan-Meier curves for **(A)** overall survival in the tumor immune microenvironment (TIME) subset, **(B–E)** overall survival by TIME features and **(F)** overall survival by LKB1 expression in the TIME subset. OS, overall survival; TC, tumor cells; TIIC tumor immune infiltrating cells.

# Supplementary Tables

# Supplementary table 1. Tumor immune microenvironment subset clinical features.

| **Variable** | **n = 70** |
| --- | --- |
| Sex |  |
| male | 48 (68.6%) |
| female | 22 (31.4%) |
| Age |  |
| median (range) | 68 (47-81) |
| < 70 | 40 (57.1%) |
| ≥ 70 | 30 (42.9%) |
| Smoke |  |
| yes (current/former) | 67 (95.7%) |
| no | 3 (4.3%) |
| ECOG PS |  |
| ≤ 1 | 70 (100.0%) |
| > 1 | 0 (0.0%) |
| Symptoms at diagnosis |  |
| yes | 47 (67.1%) |
| no | 23 (32.9%) |
| Weight loss at diagnosis |  |
| yes | 10 (14.3%) |
| no | 60 (85.7%) |
| Stage at diagnosis |  |
| limited | 54 (77.1%) |
| extended | 16 (22.9%) |
| CNS metastases at diagnosis | 6 (37.5%) |

ECOG PS, Eastern Cooperative Oncology Group performance status; CNS, central nervous system; ChT, chemotherapy; RT, radiotherapy.

# Supplementary table 2. Tumor immune microenvironment features.

| **Variable** | **n = 70** |
| --- | --- |
| CD8+ TILs |  |
| 0-2+ | 61 (87.1%) |
| 3+ | 9 (12.9%) |
| FOXP3+ TILs |  |
| 1-3+ | 54 (77.1%) |
| 0 | 16 (22.9%) |
| PD-L1 TIIC |  |
| 0% | 47 (67.1%) |
| ≥ 1% | 23 (32.9%) |
| PD-L1 TC |  |
| 0% | 35 (50.0%) |
| ≥ 1% | 35 (50.0%) |

TILs, tumor infiltrating lymphocytes, TIIC, tumor immune infiltrating cells, TC, tumor cells.

# Supplementary table 3. Association between tumor immune microenvironment features and clinical features. (A) CD8+ tumor infiltrating lymphocytes. (B) FOXP3+ tumor infiltrating lymphocytes. (C) PD-L1 on tumor cells. (D) PD-L1 on tumor infiltrating immune cells.

**A**

| **Variable** | **CD8+ TILs 0-2+**  **n = 61** | **CD8+ TILs 3+**  **n = 9** | **p** |
| --- | --- | --- | --- |
| Sex |  |  | 0.4 |
| male | 43 (89.6%) | 5 (10.4%) |  |
| female | 18 (81.8%) | 4 (18.2%) |  |
| Age |  |  | 0.7 |
| < 70 | 34 (85.0%) | 6 (15.0%) |  |
| ≥ 70 | 27 (90.0%) | 3 (10.0%) |  |
| Smoke | 58 (86.6%) | 9 (13.4%) | >0.9 |
| Symptoms at diagnosis | 40 (85.1%) | 7 (14.9%) | 0.7 |
| Weight loss at diagnosis | 8 (80.0%) | 2 (20.0%) | 0.6 |
| Stage at diagnosis |  |  | 0.2 |
| limited | 49 (90.7%) | 5 (9.3%) |  |
| extended | 12 (75.0%) | 4 (25.0%) |  |

TILs, tumor infiltrating lymphocytes

**B**

| **Variable** | **FOXP3+ TILs 1-3+**  **n = 54** | **FOXP3+ TILs 0**  **n = 16** | **p** |
| --- | --- | --- | --- |
| Sex |  |  | 0.2 |
| male | 35 (72.9%) | 13 (27.1%) |  |
| female | 19 (86.4%) | 3 (13.6%) |  |
| Age |  |  | 0.3 |
| < 70 | 29 (72.5%) | 11 (27.5%) |  |
| ≥ 70 | 25 (83.3%) | 5 (16.7%) |  |
| Smoke | 52 (77.6%) | 15 (22.4%) | 0.5 |
| Symptoms at diagnosis | 34 (72.3%) | 13 (27.7%) | 0.2 |
| Weight loss at diagnosis | 8 (80.0%) | 2 (20.0%) | >0.9 |
| Stage at diagnosis |  |  | >0.9 |
| limited | 42 (77.8%) | 12 (22.2%) |  |
| extended | 12 (75.0%) | 4 (25.0%) |  |

TILs, tumor infiltrating lymphocytes

**C**

| **Variable** | **PD-L1 TIIC 0**  **n = 47** | **PD-L1 TIIC ≥ 1**  **n = 23** | **p** |
| --- | --- | --- | --- |
| Sex |  |  | 0.3 |
| male | 34 (70.8%) | 14 (29.2%) |  |
| female | 13 (59.1%) | 9 (40.9%) |  |
| Age |  |  | 0.14 |
| < 70 | 24 (60.0%) | 16 (40.0%) |  |
| ≥ 70 | 23 (76.7%) | 7 (23.3%) |  |
| Smoke | 44 (65.7%) | 23 (34.3%) | 0.5 |
| Symptoms at diagnosis | 32 (68.1%) | 15 (31.9%) | 0.8 |
| Weight loss at diagnosis | 8 (80.0%) | 2 (20.0%) | 0.5 |
| Stage at diagnosis |  |  | 0.2 |
| limited | 34 (63.0%) | 20 (37.0%) |  |
| extended | 13 (81.3%) | 3 (18.8%) |  |

TIIC, tumor immune infiltrating cells

**D**

| **Variable** | **PD-L1 TC 0**  **n = 35** | **PD-L1 TC ≥ 1**  **n = 35** | **p** |
| --- | --- | --- | --- |
| Sex |  |  | 0.12 |
| male | 21 (43.8%) | 27 (56.3%) |  |
| female | 14 (63.6%) | 8 (36.4%) |  |
| Age |  |  | 0.053 |
| < 70 | 16 (40.0%) | 24 (60.0%) |  |
| ≥ 70 | 19 (63.3%) | 11 (36.7%) |  |
| Smoke | 32 (47.8%) | 35 (52.2%) | 0.2 |
| Symptoms at diagnosis | 25 (53.2%) | 22 (46.8%) | 0.4 |
| Weight loss at diagnosis | 5 (50.0%) | 5 (50.0%) | >0.9 |
| Stage at diagnosis |  |  | 0.3 |
| limited | 25 (46.3%) | 29 (53.7%) |  |
| extended | 10 (62.5%) | 6 (37.5%) |  |

TC, tumor cells

# Supplementary table 4. Survival analysis in the tumor immune microenvironment subset.

| **Variable** |  | **mOS, months (95%CI)** | **p (log-rank)** | **Univariate HR (95%CI)** | **p** | **Multivariate HR (95%CI)** | **p** |
| --- | --- | --- | --- | --- | --- | --- | --- |
| Sex | male | 53.2 (20.8-72.4) | 0.159 | 0.68 (0.39-1.17) | 0.162 | - |  |
|  | female | 24.9 (13.0-107.1) |  |  |  |  |  |
| Age | ≥ 70 | 24.1 (8.9-93.3) | 0.913 | 0.97 (0.58-1.63) | 0.915 | - |  |
|  | < 70 | 42.5 (22.4-72.4) |  |  |  |  |  |
| Weight loss at diagnosis | no | 44.5 (30.3-68.4) | **0.026** | 0.47 (0.23-0.93) | **0.029** | 0.50 (0.24-1.00) | 0.05 |
|  | yes | 9.1 (1.1-NC) |  |  |  |  |  |
| Symptoms at diagnosis | no | 70.3 (60.2-120.1) | **0.047** | 0.58 (0.34-0.99) | **0.049** | 0.90 (0.49-1.64) | 0.73 |
|  | yes | 14.7 (8.1-33.6) |  |  |  |  |  |
| PD-L1 TC | 0% | 31.7 (13.0-70.3) | 0.807 | 1.07 (0.63-1.82) | 0.81 | - |  |
|  | ≥ 1% | 33.6 (22.4-82.1) |  |  |  |  |  |
| PD-L1 TIIC | 0% | 20.8 (11.9-62.3) | 0.158 | 1.44 (0.87-2.39) | 0.159 | - |  |
|  | ≥ 1% | 53.2 (31.7-109.3) |  |  |  |  |  |
| CD8+ TILs | 0-2+ | 42.5 (­­16.3-68.4) | 0.493 | 0.78 (0.38-1.59) | 0.495 | - |  |
|  | 3+ | 22.4 (4.9-NC) |  |  |  |  |  |
| FOXP3+ TILs | 0 | 24.1 (6.2-62.4) | **0.001** | 2.18 (1.19-4.00) | **0.001** | 2.07 (1.11-3.86) | **0.02** |
|  | 1-3+ | 42.5 (16.3-82.1) |  |  |  |  |  |
| LKB1 | ≤ 4 | 20.8 (7.6-70.3) | 0.062 | 1.68 (0.97-2.91) | 0.065 | - |  |
|  | > 4 | 53.2 (15.4-82.1) |  |  |  |  |  |
| Stage at diagnosis | extended | 1.8 (0.9-14.7) | **<0.001** | 6.10 (3.18-11.71) | **<0.001** | 5.28 (2.64-10.5) | **<0.001** |
|  | limited | 62.3 (32.4-81.0) |  |  |  |  |  |

HR, hazard ratio; 95%CI, 95% confidence interval; mOS, median overall survival; TILs, tumor infiltrating lymphocytes; TIIC, tumor immune infiltrating cells; TC, tumor cells.

# Supplementary Materials and Methods

| **Primary antibody** | **Catalog number** |
| --- | --- |
| LKB1 Ley 37D/G6 clone (Santa Cruz Biotechnology, Dallas, TX, USA) | sc-32245 |
| CD8 C8/144B clone (DAKO, Carpinteria, CA, USA) | IR623 |
| FOXP3 236A/E7 clone (Abcam, Cambridge, UK) | ab20034 |
| PD-L1 22C3 clone (DAKO, Carpinteria, CA, USA) | M3653 |
